# Volumetric imaging: a potential tool to stage upper tract urothelial carcinoma

**DOI:** 10.1007/s00345-019-02682-1

**Published:** 2019-02-28

**Authors:** Alexandra Grahn, Nobuyuki Tanaka, Per Uhlén, Marianne Brehmer

**Affiliations:** 1 Department of Oncology and Pathology, Karolinska Institutet, Stockholm, Sweden; 2 Division of Urology, Karolinska University Hospital, Stockholm, Sweden; 3 Department of Urology, Keio University School of Medicine, Tokyo, Japan; 4 Department of Medical Biochemistry and Biophysics, Karolinska Institutet, Solnavägen 9 (C0669), SE-171 77 Stockholm, Sweden; 5 Division of Urology, Department of Clinical Sciences, Danderyd Hospital, Karolinska Institutet, Stockholm, Sweden

**Keywords:** Volumetric imaging, Three-dimensional imaging, Urothelial carcinoma, Diagnostic accuracy, Tumor heterogeneity, Prognostic tumor marker

## Abstract

**Purpose:**

To investigate whether volumetric imaging of tumor vasculature can be used to phenotypically characterize advanced upper tract urothelial carcinoma, and if this technique can distinguish aggressive invasive tumors from non-aggressive superficial ones.

**Methods:**

In a pilot study, two TaG1 and two T3G3 formalin-fixed paraffin-embedded (FFPE) tumor samples were examined using the DIPCO pipeline (Tanaka et al. in Nature Biomed Eng 1(10):796–806. 10.1038/s41551-017-0139-0, 2017). Briefly, punch biopsies of FFPE tumors were deparaffinized, cleared, immunolabeled with the vessel marker CD34 and imaged with a light-sheet microscope. Thereafter, the three-dimensional (3D) vasculature of the tumors was analyzed and characterized using a specialized image processing software.

**Results:**

We found that T3G3 tumors had increased CD34 density kurtosis and skewness compared to TaG1 tumors. This suggests that analysis of the 3D vasculature can distinguish between high-grade invasive and low-grade superficial tumors.

**Conclusions:**

Volumetric imaging of tumor samples may represent novel methodology that can complement conventional histopathology. Volumetric imaging enabled us to differentiate between invasive and non-invasive upper tract urothelial carcinoma. The method is of particular interest in diagnostic work-up of patients with upper tract urothelial carcinoma as previous findings indicate that volumetric imaging of vascular patterns could be used to differentiate superficial and invasive urothelial carcinoma, irrespective of if the tumor sample was deep or superficial. However, further and more extensive studies are required before this method can be applied clinically.

## Introduction

Upper tract urothelial carcinoma (UTUC) is a rare but lethal malignancy. Invasive UTUC (pT2/pT3) has a 5-year disease-specific survival of < 50% [2]. Nephroureterectomy (NU) is the gold standard for treatment of organ confined high-risk UTUC. In low-risk disease, kidney-sparing surgery (KSS) and NU offer similar disease-specific survival [3]. The key issue in diagnostic work-up of UTUC is to distinguish between patients with low-risk disease that can safely benefit from KSS, and those who are at risk of more invasive disease and require prompt radical NU. Stratification into low- and high-risk tumors [2] may aid choice of treatment modality (i.e., radical or KSS), but it is a matter of debate what parameters are essential for prognostic assessment. Recent studies suggest that tumor size and multifocality do not affect prognosis and efficacy of KSS to the same degree as tumor stage and grade [4–6].

The European Association of Urology (EAU) guidelines recommend computed tomography urography (CT urography), cystoscopy, and urinary cytology for diagnostic workup. Ureteroscopy (URS) with focal samples adds diagnostic accuracy and is necessary when KSS is an option [2, 7].

Stage and grade are the strongest prognostic factors recurring in the literature [2, 6, 8–10]. Stage is difficult to determine when examining small ureteroscopic biopsies [11] with conventional 2D microscopes [7, 8], but fortunately there is good correlation between stage and grade [9]. Nonetheless, correct grading has proven to be challenging [12–14], and under-grading is a major concern when KSS is considered. Further, histologic findings in a biopsy are representative only of the location where the tissue specimen was collected. Smith et al. [12] reported that one-third of their patients showed a change in stage or grade between initial diagnostic and repeat biopsies (median interval 6 weeks). This observation might be explained by intratumoral heterogeneity, resulting in disparate histological features in different areas of the tumor. In situ barbotage cytology can help increase diagnostic accuracy [15], but other more reliable methods are needed to differentiate between non-aggressive superficial and aggressive invasive UTUC. Prognostic biomarkers have been evaluated in several studies, but none are yet in general wide clinical use [16].

Tanaka et al. [1] presented a novel technique that uses volumetric imaging to investigate tumor phenotype. With this approach it is possible to analyze three-dimensional (3D) structures (e.g., the vasculature and cell niches) within a volume of a tumor sample, and thereby add depth to conventional two-dimensional (2D) histology. Tanaka et al., named this method DIPCO (diagnosing immunolabeled paraffin-embedded cleared organs) and used it to investigate large biopsy specimens from urothelial carcinoma, mainly from the bladder. In addition to disparities in phenotype within the tumors, these authors found that vascular patterns differed between advanced urothelial carcinoma and superficial tumors. Assessment of the DIPCO method revealed that volumetric imaging was more accurate than 2D imaging in predicting the invasiveness of muscle invasive bladder cancer [1]. In another study with similar experimental set-up, Tanaka et al. [17] showed that volumetric imaging could determine lymphatic system invasion with higher accuracy than standard 2D histological diagnostic methods. Further, their findings suggest that volumetric imaging of vascular patterns could be used to differentiate superficial and invasive urothelial carcinoma, regardless if the tumor sample was deep or superficial. This would be very helpful for diagnosing UTUC, as biopsies tend to be superficial.

In this pilot study, we assessed whether the DIPCO pipeline could be used to characterize UTUC, and whether analysis of the 3D structure of the vasculature could distinguish between high-grade invasive and low-grade superficial tumors.

## Methods

### Sample selection

Tumor samples were selected from a previously described consecutive cohort of UTUC patients, including patients with different grades of UTUC that had undergone NU after diagnostic URS during the period 2005–2012 [15]. We selected two TaG1 and two T3G3 tumor samples that clearly represented non-aggressive superficial and aggressive invasive tumors, respectively. The samples were formalin-fixed paraffin-embedded (FFPE) tumors that were graded according to the WHO 1999 classification. One experienced uropathologist selected blocks with relevant tumor material to represent high-grade invasive and low-grade non-invasive UTUC respectively. A punch biopsy (diameter 3 mm, depth 1 mm) was taken from each sample. Three of four punch biopsies investigated did only include tumor material without tumor base and lamina propria. The fourth one, from a TaG1 tumor, slightly touched the tumor base at the edge. Normal urothelium from the bladder, from the material of Tanaka et al. [1] was used as reference.

## Sample preparation

The FFPE samples were prepared as previously described by Tanaka et al. [1]. Briefly, the samples were deparaffinized, cleared according to the iDISCO (immunolabelling-enabled three-dimensional imaging of solvent-cleared organs) protocol and immunolabeled as follows:

Deparaffinization

- Three applications of xylene for 1 h, the two first at 37 °C and the last at room temperature.
- Washing 1 h each with decreasing concentrations of ethanol (100%, 95%, 90%, 80%, 70%).
- Incubation in PBS overnight.

Clearing according to the iDISCO protocol

- Applications of increasing concentrations of methanol (20%, 40%, 60%, 80%, and 100%).
- Overnight bleaching with 5% hydrogen peroxide in 20% DMSO and methanol at 4 °C.
- Applications of decreasing concentrations of methanol (80%, 60% 40%, and 20%).
- Washing with PBS and thereafter with PBS/0.2% Triton X-100.

Whole-tissue immunolabeling

- Incubation with 20% DMSO, 0.2% Triton X-100, and 0.3 M glycine at 37 °C overnight.
- Blocking with PBS, 0.2% Triton X-100, 10% DMSO, and 6% donkey serum at 37 °C for 1 day.
- Washing with PBS and 0.2% Tween-20.
- Incubation with primary antibody anti-CD34 (1: 100, mouse monoclonal, no. ab8536, Abcam) in PBS, 5% DMSO, and 3% donkey serum at 37 °C for 4 days.
- Incubation with secondary antibody (1: 200) Alexa 647-conjugated affinity purified F(ab’)2 fragment donkey anti-mouse IgG H + L antibody (no. # 715–606-151, Jackson ImmunoResearch Laboratories, West Grove, PA, USA) at 37 °C for 4 days.
- Washing with PBS and 0.2% Tween-20 for 2 days.

## Microscopy

To visualize tumor samples, we used a custom-built light sheet microscope equipped with a 10× objective lens (NA 0.6, XLPLN10XSVMP, Olympus) and an sCMOS camera (Hamamatsu ORCA-Flash4.0). The samples were placed in a pure quartz cuvette (Starna Cells) filled with dibenzyl ether (DBE). The xy resolution was 0.585 µm, and the z-step was 5 µm. The data were downsized to 5 × 5 × 5 µm^3^ before 3D image-processing.

## Sample analysis

The vascular network in the four tumor samples were analyzed without physically cutting the samples into sections. Instead, we digitally split them into smaller regions: for each 5-μm z-section the CD34 density was calculated using Amira software. The number of regions, i.e., the dataset for calculating the heterogeneity features (kurtosis, skewness, and variance of CD34 density) varied between 377 and 506, see ‘‘Results”. The CD34 density was quantified by calculating the percentage CD34 immunosignal in the different regions. The intratumoral vessels were identified using an intensity-based threshold (hysteresis thresholding). CD34-positive regions were then interconnected by a software tool in Amira to segment and visualize vascular networks in the tissue [1]. The spatial graph view algorithms within the 'Auto Skeleton' function of the Amira software (from FEI) calculated vessel thickness, length and radius. The authors who performed the laboratory work, microscopy, and initial data analyses were blinded to tumor grade and stage. We did not analyze significance due to the small number of samples.

In probability theory and statistics, kurtosis describes the shape of the probability distribution, and skewness is a measure of the asymmetry of the probability distribution. The kurtosis and skewness were used to quantitatively characterize the vascular network in the tissue, assessing distinct features, i.e., vascular patterns, between superficial and aggressive tumors.

## Results

**Fig. 1:**
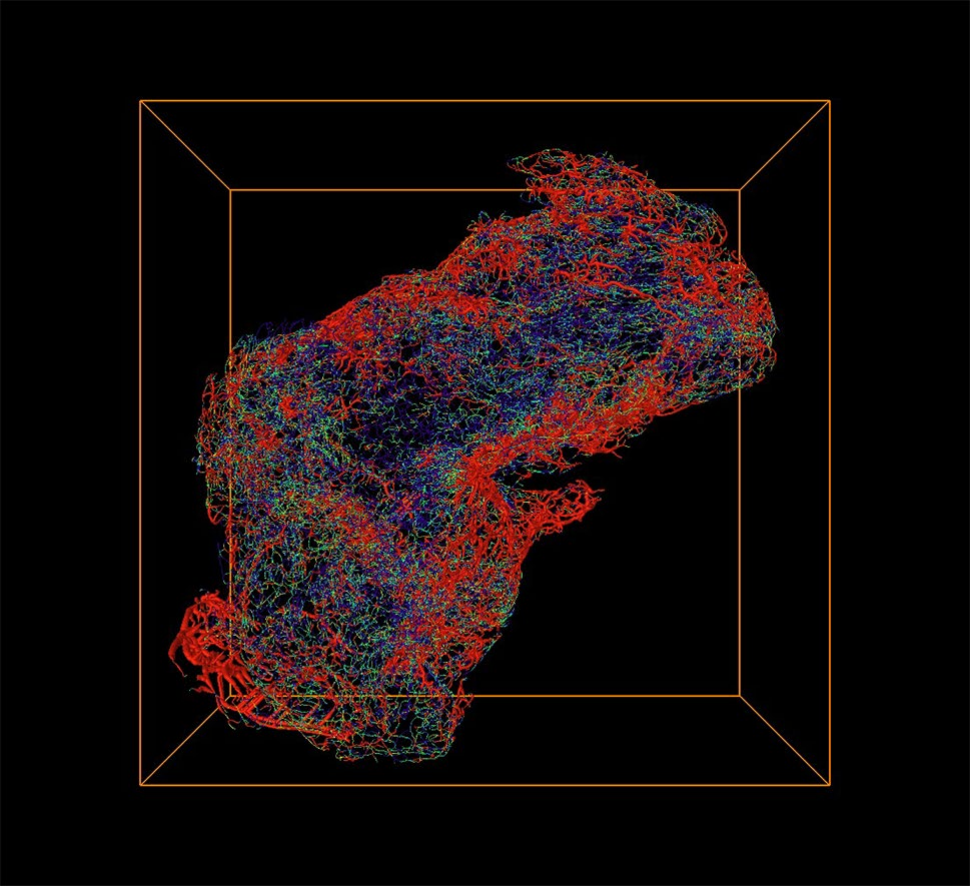
Spatial graph view generated by Amira software (from FEI) of the 3D vascular network in a T3G3 (high-grade invasive) tumor, visualizing the heterogeneity in the vascular network. The vessel radius is indicated by pseudo-coloring: red indicates thick vessels (> 11 μm), green indicates intermediate vessels, and blue indicates thin vessels (< 3 μm)

Two superficial grade 1 (TaG1) tumors and two invasive grade 3 (T3G3) tumors were immunostained with the vessel marker CD34 and examined using the DIPCO pipeline. We evaluated the heterogeneity of the blood vessels by applying an advanced 3D image-processing algorithm to study vessel thicknesses (see “Methods”). Volume rendering of the results of this analysis revealed an intertwined vascular network with complex 3D structures (see Fig. 1).

We subsequently determined whether DIPCO could stratify the aggressiveness of our four tumor samples. We quantified CD34 density, vessel radius, and heterogeneity features (kurtosis, skewness, and variance of CD34 density; see “Methods”). The heterogeneity features were calculated per tumor sample, based on a dataset of 483 and 377 measurements in tumor samples 1–2 (TaG1), and 506 and 414 measurements in tumor samples 3–4 (T3G3). This analysis revealed that the CD34 density kurtosis and skewness were markedly higher in the T3G3 lesions than in the TaG1 tumors (see Table 1). Both tumor types differed distinctly in these parameters from normal urothelium. There were small differences in CD34 density, which increased slightly with tumor aggressiveness, and vessel radius which was slightly higher in the superficial tumors.

**Table 1:** Characteristics of the immunosignal level of CD34 in the four different tumors, two TaG1 tumors and two T3G3

Sample number	Tumor stage and grade	CD34 density	Variance	Kurtosis	Skewness	Vessel radius (µm)
1	TaG1	25.67	0.001135	2.870	1.657	3.579
2	TaG1	13.96	0.02158	4.865	0.5529	3.118
Mean TaG1	TaG1	19.81	0.01135	3.867	1.105	3.349
3	T3G3	22.51	0.006638	16.09	3.734	3.197
4	T3G3	21.65	0.01466	23.01	3.845	2.733
Mean T3G3	T3G3	22.08	0.01065	19.55	3.790	2.965
Mean normal urothelium		17.27	0.0006380	– 0.2058	0.3302	2.998

The CD34 density kurtosis and skewness were markedly higher in the T3G3 lesions than in the TaG1 tumors. However, both tumor types differed markedly from normal urothelium in these parameters. Tumor stage and grade are classified according to the WHO 1999 classification. Variance, kurtosis and skewness are referring to CD34 density

## Discussion

This pilot study of the vascular patterns of UTUC demonstrated that compared to the low-grade superficial tumors, the high-grade invasive tumors showed increased kurtosis and skewness of CD34 density. These results indicate that high-grade invasive tumors have more heterogeneous non-structured vascular networks than low-grade tumors and normal urothelium. We also found that the mean CD34 density increased slightly with tumor aggressiveness and that vessel radius was slightly higher in the superficial tumors; however, these differences were small and must be assessed in a larger cohort. To some extent, our data correlate with the findings of Tanaka et al. [1] who showed that a multi-parameter model incorporating vessel radius and CD34 density kurtosis could differentiate between muscle-invasive and non-invasive bladder tumors. Tanaka et al., stratified their material according to stage. Since our cohort was limited, we did not carry out such stratification. Our results should be seen as dependent on a combination of stage and grade, tumor characteristics with a close correlation [9].

Tanaka et al., also analyzed CD34 density skewness and noted a significant disparity between normal tissue and < pT2 bladder tumors. Tanaka and coworkers primarily investigated urothelial carcinoma of the bladder (UCB), and previous research has shown partial similarity between UTUC and UCB. Sfakianos et al. [18] studied genetic aberrations in UTUC and UCB in 300 cancer-associated genes, and those authors concluded that although there are many similarities in gene mutations, there are also many differences between UCB and UTUC.

Our findings of more aberrant vasculature in high-grade invasive tumors clearly agree with the literature on tumor vascularity. Hanahan and Weinberg [19] noted that the vascular architecture tends to be abnormal in tumors as compared to normal tissue. Furthermore, Weinberg [20] observed that increased angiogenesis is usually correlated with more aggressive tumor phenotype and worse clinical outcome in many cancer types. Additionally, separate studies performed by Nakanishi et al. [21] and Ke et al. [22] both found correlations between increased expression of HIF-1, a hypoxia-induced driver of angiogenesis, and poorer prognosis and lower survival in UTUC patients.

Under-grading of ureteroscopic biopsies is a major concern. It is difficult to obtain a ureteroscopic biopsy with sufficient depth to analyze invasiveness without perforating the thin urethral wall. Tanaka et al., selected a single random tissue block from each patient in their study, i.e., each sample originated from either a superficial part of the tumor or an area close to the tumor base. Nevertheless, those investigators still saw patterns of vascularity that, similar to those we observed, exhibited distinct differences between invasive and superficial urothelial carcinoma. Three out of four tumor samples in our study did not include tumor base, but yet our results could distinguish the aggressive invasive tumors from the non-invasive non-aggressive ones, as well as the tumors from normal bladder tissue. Our findings suggest that to diagnose invasiveness, volumetric imaging may be less sensitive to where a tumor sample is taken than conventional histopathology, which would be very helpful in diagnosing UTUC, as biopsies tend to be superficial.

Our results are consistent with Tanaka et al., and many other reports in the field showing a significance of vasculature in tumors. However, our cohort was limited, and hence our data should be seen as hypothesis-generating. To be of true clinical value, our findings must be assessed in a prospective pretreatment setting, possibly by means of volumetric imaging of fresh URS biopsies. Normal urothelium from the upper urinary tract should be used as reference, preferably from patients without UTUC. Another very interesting application would be to explore whether this method can differentiate between superficial and invasive G2 tumors and/or Ta low-grade and Ta high-grade UTUC.

## Conclusion

Our results suggest that volumetric imaging of tumor volumes can be a valuable tool to complement conventional histopathology when differentiating between high-grade invasive UTUC and low-grade superficial tumors. This imaging method could aid treatment decisions in UTUC.
